# Opportunistic random searcher versus intentional search image user

**DOI:** 10.1038/s41598-018-21563-y

**Published:** 2018-02-20

**Authors:** József Garay, Zoltán Varga, Tamás F. Móri, Inmaculada López, Manuel Gámez, Juan R. Gallego, Tomás Cabello

**Affiliations:** 1 MTA-ELTE Theoretical Biology and Evolutionary Ecology Research Group and Department of Plant Systematics, Ecology and Theoretical Biology, Eötvös Loránd University, Pázmány Péter sétány1/c, H-1117 Budapest, Hungary; 2 MTA Centre for Ecological Research, Evolutionary Systems Research Group., Klebelsberg Kuno utca 3, Tihany, 8237 Hungary; 3 Department of Mathematics, Szent István University, Páter K. u. 1.H-2103, Gödöllő, Hungary; 4 Department of Probability Theory and Statistics, L. Eötvös University, Pázmány Péter sétány1/c, H-1117 Budapest, Hungary; 5 Department of Mathematics, University of Almería. La Cañada de San Urbano, 04120 Almería, Spain; 6 Center for Agribusiness Biotechnology Research, Almería University, Ctra. Sacramento s/n, ES-04120 Almería, Spain

## Abstract

We consider two types of optimal foragers: a random searcher and a search image user. A search image user can find its desired prey with higher and undesired prey with lower probability than a random searcher. Our model considers the density-dependent travelling time and the time duration of reproduction (oviposition). In the framework of optimal foraging theory for one predator–two prey systems, we find that there are ranges of prey densities in which the search image user has a higher net energy intake, and there are other ranges of prey densities in which the random searcher has higher net energy intake. The damsel bug *Nabis pseudoferus* Remane (Hemiptera: Nabidae) is a generalist predator rather than an omnivore. This species has a wide range of arthropod prey (predominantly insects and mites). Several aspects of the biology of this species have been studied, especially its cannibalistic behaviour, which is a quite important feature because *N*. *pseudoferus* is often used as a biological control agent against lepidopteran pests in greenhouse crops. Experimentally, we found that *Nabis* is a search image user in the above sense.

## Introduction

In this paper, we are interested in identifying the effect of the search image^1–6^ on optimal foraging. According to Tinbergen^1^, the search image is a perceptual change that improves the predator’s ability to detect its desired prey type. Bond and Riley^4^ introduced an accumulator model of visual search that includes discriminability (i.e., the focus on visual features that are characteristic of a particular item), response bias (i.e., an increased predisposition to respond to food-related stimuli), and a “caution” hypothesis (i.e., attributing improvements in stimulus detection to changes in the amount of evidence that the animal acquires before making a response). We use the phenomenological definition of a search image provided by Dukas^7^, i.e., a “*selective search for a particular cryptic prey type*, *which involves an increased probability of detecting that prey type and a reduced probability of detecting other distinct prey types”*. Note that a search image implies a trade-off between encounters with preferred and non-preferred prey types. In our words, the *search image user* (**SIU**) can find its desired prey with higher probability and its undesired prey with lower probability than a random searcher. In the standard optimal foraging models, the forager is a *random searcher* (**RS**), i.e., its prey preference does not affect the encounter probabilities with the prey types; in other words, the encounter probabilities are determined only by the preys’ densities.

In predation, the encounter is one of the most important steps^8,9^. Finding the prey is a complicated stochastic process^10^, and the encounters are determined at least by two main factors: the perception ability of the predator^11^ and the prey densities^12^. Here, we will consider the case in which the predator finds its desired prey with a high, density-independent probability, but the travelling time^13^ of the predator will be longer at lower prey densities. In a one predator–two prey system, the question arises: Which has higher net energy intake, the **SIU** or the **RS**?

We note that in greenhouses, these systems often occur. The prey preference of the agent is important in biological control. For instance, if the agent’s preferred prey causes less damage than the non-preferred prey, then the economic efficiency of the agent is not optimal^14,15^. The basic picture is motivated by the behaviours of certain predatory insects that practically forage continuously with the exception of egg laying. For example, *Nabis pseudoferus* Remane displays such behaviour^16^. *Nabis* eats all day; hence, the time required to lay eggs reduces the time available for predation. The latter fact, in particular, will play an important role in the calculation of the numerical response. The female *Nabis* is territorial and uses visual and odour stimuli during hunting^16^. *Nabis pseudoferus* can be considered to be a non-omnivorous predator^17^. The majority of studied Nabidae species also practice plant feeding, but they are not able to develop in the absence of prey^18–20^. The plant feedings only serve to search for water sources and seem to do little or no damage to the plant^21^. This practice seems to help the predator to survive during prey scarcity^20^. *N*. *pseudoferus* has a wide range of prey and has been cited as an important predator of aphids^22,23^ but is also a voracious predator of lepidopterans and other groups of arthropods, including hemipterans and mites^24–27^. *N*. *pseudoferus* is, from the trophic perspective and according to Hurd^28^, a strict predator with a wide range of prey arthropods. Several aspects of the biology of this species have been studied. Thus, it is known, on the one hand, that adults and nymphs of *N*. *pseudoferus* exhibit a type II functional response. In general, other *Nabis* species also present type II responses in their nymph and adult stages^29^. On the other hand, cannibalism in *N*. *pseudoferus* has also been documented and is an aspect of great importance in the biology of this species^29^ because *N*. *pseudoferus* is also used as a biological pest control agent of lepidopterans in greenhouses crops^30,31^.

The paper is organized as follows: In a Theoretical study, we first calculate the numerical response when reproduction and travelling also require time. We compare the per-unit time energy intakes of **RS** and **SIU**. In the Experimental study, based on our laboratory trials, we test whether the *Nabis* uses search images. The mathematical details, the experimental data and a new statistical method are summarized in the Supplementary Information (SI).

## Theoretical Study

### Assumptions

In a habitat of area *M*, there are *H* perception ranges (PR-s), and in the habitat, there are two types of prey: *x* is the number of A-prey, *y* is the number of B-prey, and we suppose that *x* + *y* < *H*. Assume that the habitat is homogeneous^12,32^, i.e., in all PR-s, the predator-prey interactions are the same. For example, we can consider the following situation: A predator insect searches for prey on a given plant, and the perception ranges are the leaves.

For simplicity, we have assumed that the prey exhibit no anti-predator behaviours, i.e., the predator can kill any encountered prey. Specifically, both of the prey can neither defend against the predator nor flee^33^, they do not have gregarious behaviour^34^, and there is no refuge^35^. The above simplifying assumptions imply that the prey have no effect on predation; therefore, we will have an optimal foraging model in which the predator maximizes its numerical response. Each perception range contains, at most, one prey, so the prey types are randomly separated in the PR-s in the home range of the predator. There is a stationary distribution of perception range types, PR:X (X = E (empty), A, B), which does not change during time *T*, e.g., one day.

The optimal forager predator has territory, thus, there is no interaction between two predators during hunting^36–38^. For simplicity, we also assume that there is no nutritional difference between the different prey types with the exception of energy content^39^. The searching processes of the predator and the distribution of the prey are independent. The travelling time required to find a prey depends on the density of the prey, i.e., it is longer at lower prey densities. An **RS** visits the nearest perception range, and the random distributions of prey ensure the random encounters. An **SIU** uses a search image and finds its desired prey type with a density-independent probability. We will compare the numerical responses of these two types of predators.

### What is the numerical response if reproduction also requires time?

In optimal foraging theory, a widely used assumption is that the numerical response equals the functional response weighed with a conversion coefficient. However, if reproduction (oviposition or offspring care) also requires time (similar to searching for prey and the handling of prey), and reproduction and hunting exclude each other, then the numerical response and the functional response are not V proportional. We emphasize the assumption that the time durations of the predator’s activities do not overlap, which is one of the basic requirements for the derivation of functional responses^8,13^. We note that, in the case of offspring care by females, males and females would have different functional responses; e.g., female crocodiles strongly defend her nets against territorial predators, and thus, during the hatching period, the female crocodile has a lower functional response. Similarly, male emperor penguins (*Aptenodytes forsteri*) do not hunt during the hatching season.

For *Nabis*, during the time period *T*, the predator either predates (*T*_*P*_ denotes the total time duration of predation during *T*) or lays eggs (*T*_*E*_ is the total time duration of laying eggs during *T*). Reproduction and predation exclude each other; thus, we have *T* = *T*_*P*_ + T_E_. The number of eggs, however, also depends on the collected energy, so *T*_*P*_ and *T*_*E*_ are not independent. Based on the energy balance for the time period *T*, in **SI.1**, we calculate the numerical response:

$$W({\bf{s}})=\frac{E({\bf{s}})-{E}_{CL}}{E({\bf{s}}){t}_{E}+{E}_{E}},$$

where *E*(**s**) is the energy intake in unit time by a predator using foraging strategy **s**, *E*_*CL*_ is the cost of living of a female predator in unit time, *E*_*E*_ is the energy cost of one egg (including both the searching cost for a good place for the egg and the energy cost of egg laying), and *t*_*E*_ is the time duration of laying one egg, *t*_*E*_ > 1. Observe that the numerical response is a strictly increasing function of the energy intake in the unit time of predation. Specifically, the numerical response and the energy intake will reach their maxima with the same foraging strategy.

### Searching time and travelling time

Our basic assumption is that, regardless of the PR type that the searching predator finds, it will be the nearest one from that type. The searching time *τ*_*S*_ has two components: the first is travelling time *τ*_*TX*_ (X = E, A, B), which depends on the densities of A-prey and B-prey, and the second is the local searching time *τ*_*LS*_ in the PR. For simplicity, we assume that *τ*_*LS*_ does not depend on the contents of the focal PR. Thus, we have *τ*_*S*_ = *τ*_*TX*_ + *τ*_*LS*_. In **SI.2** we prove the following statements. If type PR:X has a density *λ*_*X*_, and is randomly distributed, then the average distance between the nearest PR:X and the predator is the following: In one dimension (for a predator moving along a straight line), the average distance is $\frac{1}{2{\lambda }_{X}}$, in two dimensions (for a predator moving along a plane), the average distance is $\frac{1}{2{\lambda }_{X}^{1/2}}$, and in 3 dimensions, the average distance is approximately $0.55396{\lambda }_{X}^{-1/3}$. We emphasize that the dimension of the travelling mode of the predator has an important effect on the functional response^40^.

Now we are in a position to calculate the optimal foraging strategy for each of the two types of predators.

### The random searcher does not use a search image

**Figure 1:**
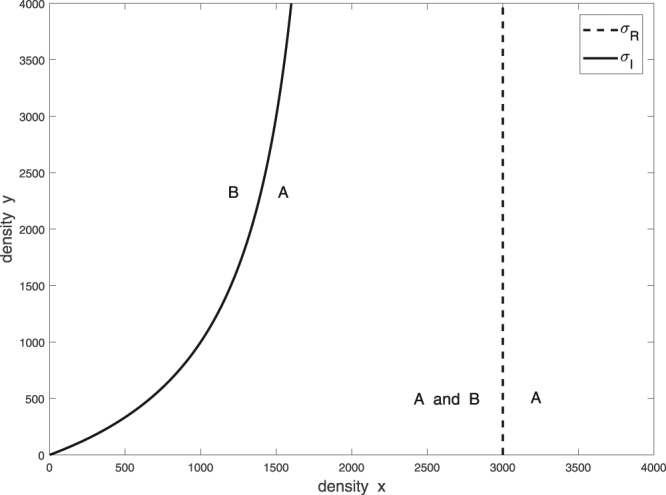
Switching curve σ_*R*_ separating the density ranges in which the **RS** eats only A (right side) and eats A and B (left side). Switching curve σ_*I*_ separating density ranges in which the **SIU** searches for A (right side) and searches for B (left side).

The **RS** is similar to a forager in the standard optimal forging model^41^. Because the prey are randomly distributed, the encounter sequence of the **RS** is random, i.e., the prey preference of the **RS** does not modify the encounter probabilities. However, we have two novel points: the **RS** looks for the nearest PR, so its travelling time depends on the density of the PR. After an encounter with a given prey type, the RS, as an optimal forager, accepts or ignores the encountered prey type, so the sequences of encounters and the sequences of killings may be different. Moreover, the **RS**’s numerical response also depends on the oviposition time. As mentioned above, the numerical response will reach its maximum with the same strategy that maximizes the average energy intake; thus, the **RS** applies the well-known zero-one rule^41^, namely,

$${s}_{A}^{\ast }=1,{\rm{and}}\,{s}_{B}^{\ast }=\{\begin{array}{c}0,{\rm{if}}\,x > \frac{{\tau }_{S}{c}_{B}}{{c}_{A}{\tau }_{B}-{c}_{B}{\tau }_{A}}H\\ 1,{\rm{if}}\,x < \frac{{\tau }_{S}{c}_{B}}{{c}_{A}{\tau }_{B}-{c}_{B}{\tau }_{A}}H\end{array},$$

where *c*_*A*_, *c*_*B*_ are the energy contents, and *τ*_*A*_*, τ*_*B*_ are the handling times of A-prey and B-prey, respectively (Fig. 1, for mathematical details see **SI.3**). In the usual sense, we say that A is more valuable than B, if $\frac{{c}_{A}}{{\tau }_{A}} > \frac{{c}_{B}}{{\tau }_{B}}$. When the more valuable prey type is abundant, the **RS** will only accept this type and ignore the other type. In this case, the encounter sequence (a random run of PR-s that are empty or contain A or B prey) and killing sequence (only the more valuable prey) are different. If the more valuable prey type is sufficiently rare, then the **RS** opportunistically accepts both prey types. In this case, the encounter and killing sequences are the same, and both are random.

### Search image user

For simplicity, we assume that the **SIU** can find its nearest desired prey with a probability of 1; thus, the **SIU** cannot find an empty PR. The **SIU** has two searching modes; when looking for an A-prey, it cannot encounter a B-prey, and vice versa. Thus, this type of predator has only a one-dimensional optimal foraging strategy; it looks for an A-prey with a probability *s*, and for a B-prey with a probability 1-*s*. Furthermore, there are two density-dependent travelling times that correspond to the desired prey type: *τ*_*TA*_ and *τ*_*TB*_. In **SI.4**, we calculate the optimal foraging strategy and find that

$${s}^{\ast }=1,{\rm{if}}\,\frac{{c}_{A}}{{t}_{A}} > \frac{{c}_{B}}{{t}_{B}};{\rm{and}}\,{s}^{\ast }=0,\,{\rm{if}}\,\frac{{c}_{A}}{{t}_{A}} < \frac{{c}_{B}}{{t}_{B}},$$

where ${t}_{A}={\tau }_{TA}+{\tau }_{LS}+{\tau }_{A}$ and ${t}_{B}={\tau }_{TB}+{\tau }_{LS}+{\tau }_{B}$ are the density-dependent time durations of rounds of killing an A-prey and a B-prey, respectively. Thus, the **SIU** only accept the prey type that ensures a higher energy intake rate during the entire time period *T*. Observe that the encounter and the killing sequences of an **SIU** are the same and not random because the prey preference determines the encounters (Fig. 1).

We note that if the **SIU** can find its nearest desired prey with a probability less than but sufficiently near 1, then the **SIU** can also be opportunistic in the sense that, if looking for B-prey, it finds an A-prey, the **SIU** may also kill the A prey. Observe that the trade-off of the search image implies a trade-off between intentional and opportunist because the **SIU** has a diminished chance to be opportunistic.

We emphasize that the essential difference between the **RS** and **SIU** is that their encounter sequences are different.

### Does the search image user overperform the random searcher?

First, in Fig. 1, we visualize that the different types of predators have different optimal foraging strategies, i.e., their switching behaviours are different (for calculations see **SI.5**).

Considering several rounds of predation, the sequences of encountered prey types for the **RS** and **SIU** are different because the **RS** randomly encounters both prey types according to the prey densities. In contrast, the **SIU** encounters its preferred prey type with a higher probability. Observe that in Fig. 1, in the density range to the right of σ_*R*_, both the **SIU** and **RS** only consume the more valuable A-prey; thus the killing sequences are the same, and despite this, their encounter sequences are different.

**Figure 2:**
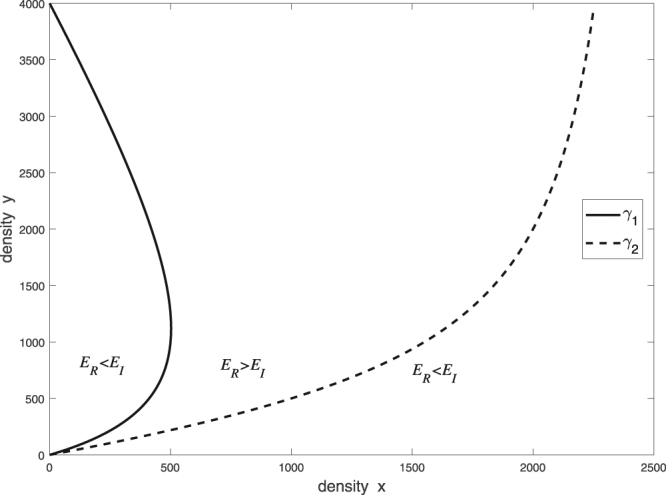
In the range between curves *γ*_1_ and *γ*_2_, the **RS** collects more energy in unit time (*E*_*R*_) than the **SIU** (*E*_*I*_). Curve σ_*I*_ in Fig. 1 would split the range *E*_*R*_ >  *E*_*I*_ into two parts.

**Figure 3:**
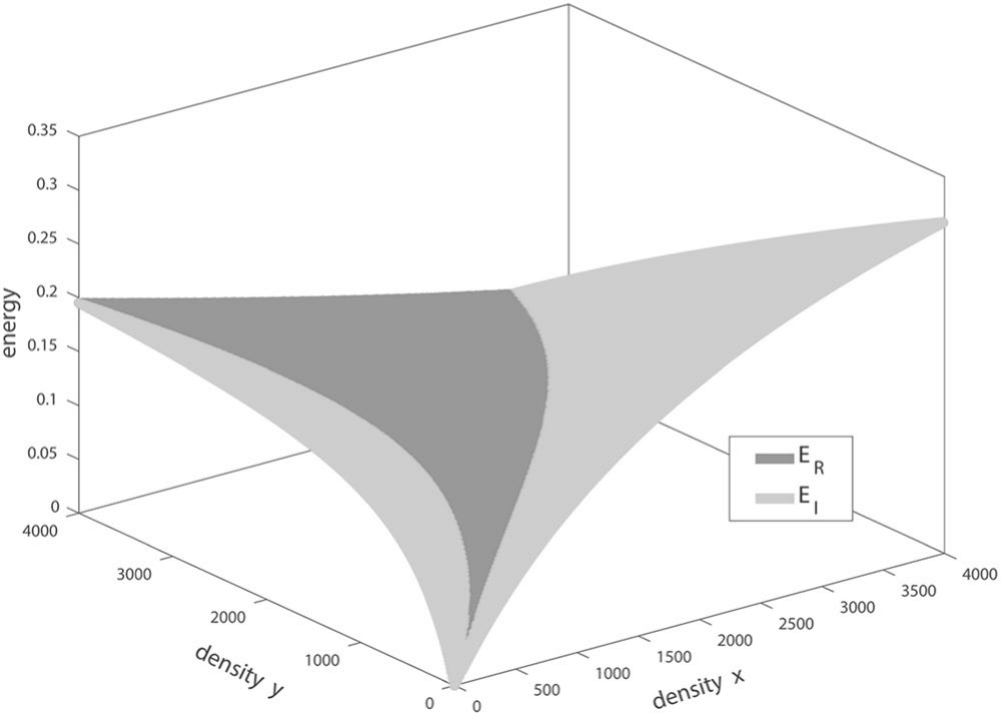
Upper hull of the energy surfaces *E*_*R*_(*x*, *y*) and *E*_*I*_(*x*, *y*). The dark area indicates the range in which the **RS** has a higher per-unit time energy intake than the **SIU**.

All these elicit the following question: Which type of predator has higher energy intake in unit time at fixed densities *x* and *y*? We found that there are two prey density ranges in which the **SIU** collects more energy than does the **RS**. Intuitively, when A-prey is scarce and B-prey is abundant, the **SIU** kills more B-prey, and the **RS** kills very few A-prey. Furthermore, if A-prey is abundant, then both predator types accept only A-prey, but the **SIU** kills more A-prey than does the **RS**. Moreover, there is a range of prey densities in which the **RS** performs better in terms of energy intake than does the **SIU** (Figs 2 and 3).

The main intuitive reason for the latter outcome is the opportunism of the **RS**^8,34^: In the range in which *E*_*R*_(*x*, *y*) >*E*_*I*_(*x*, *y*), the **SIU** kills only one type of prey, whereas the **RS** opportunistically exploits both types of prey.

Now we are in the position to provide some insight into our main question: Does the **SIU** overperform the **RS**? The answer is not necessarily. Even without setting up a concrete form of the corresponding population dynamics, we have the following two main cases:

First, assume that the population dynamics of the three-species system has a stable equilibrium. If the equilibrium prey densities lie in the range in which the **SIU** has a higher energy intake, then the **SIU** overperforms the **RS**. If the equilibrium lies in the range in which the **RS** has a higher energy intake, then the **RS** overperforms the **SIU**.

Second, assume that the population dynamics of the three-species system has no stable equilibrium, but, e.g., there is a cyclic coexistence in which the cycle touches all types of prey density ranges. Then, the optimal foraging strategy will be a mix; the predator uses either a search image or a random search according to the current prey type densities.

**Figure 4:**
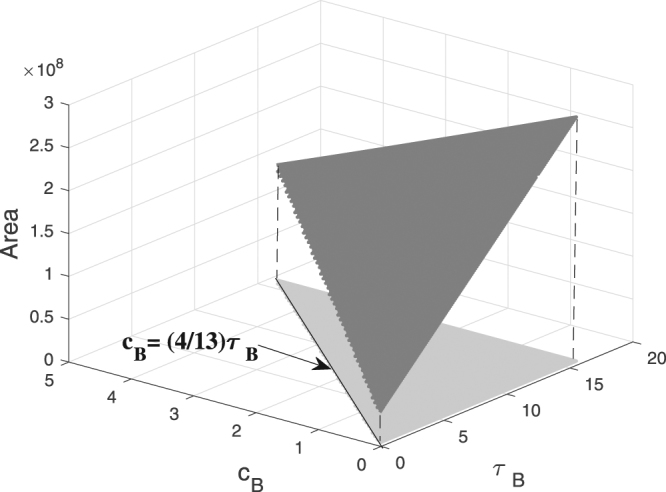
The area of the density range between curves *γ*_1_ and *γ*_2_ is strictly positive for $\frac{{c}_{B}}{{\tau }_{B}} < \frac{4}{13}$, which indicates that the higher efficiency of the **RS** is robust against changes in the parameters τ_*B*_ and *c*_*B*_.

Finally, we note that a special sensitivity analysis reveals that the density range in which the **RS** overperforms the **SIU**, is robust against changes in key parameters. Indeed, Figs 1–3 correspond to parameter choices ${c}_{A}:=1$, ${c}_{B}:=2$, ${\tau }_{LS}:=1$, ${\tau }_{A}:=1$, and ${\tau }_{B}:=8$. In our study, it is assumed that A-prey is more valuable than B-prey; formally, $\frac{{c}_{A}}{{\tau }_{A}}=1 > \frac{{c}_{B}}{{\tau }_{B}}$. In **SI.5**, a simple calculation demonstrates that, if the gap between the values of A-prey and B-prey is sufficiently large, namely, $\frac{{c}_{B}}{{\tau }_{B}} < \frac{4}{13}$, then curves *γ*_1_ and *γ*_2_ display a pattern similar to that in Fig. 2. Indeed, in this case, measuring the size of the density range in which *E*_*R*_ > *E*, with the area of the range between *γ*_1_ and *γ*_2_, indicates that for $\frac{{c}_{B}}{{\tau }_{B}} < \frac{4}{13}$, this area remains strictly positive as illustrated in Fig. 4.

## Experimental study: Results

We have demonstrated that *Nabis* uses a search image. *Nabis* is territorial, thus, females can mainly cannibalize their own offspring. Therefore, when cannibalism is rampant, it is reasonable to assume that, for the territorial cannibal predator, it is more beneficial to be an SIU that focuses on the non-conspecific prey than a RS because filial cannibalism can decrease the fitness of a female. In a Petri dish arena, *Nabis* uses visual and odour stimuli while searching and hunting^16^. *Nabis* can recognize its found possible prey by touching it with her antenna^42^. Thus, we can define an encounter by an antenna touch of an item by *Nabis*. Furthermore, we observe that *Nabis* is an opportunistic predator in the sense that if it encounters a prey, it always kills that prey. Therefore, in our case, *Nabis*’ killing sequence and its encounter sequence are the same; thus, we can use the killing sequence as the encounters sequence. As we emphasized, the existence of a search image can be detected by the non-randomness of encounter sequences, i.e., we can say that if the encounter sequence is not random, then the forager uses a search image. First, we examined the randomness of the encounter sequence of *Nabis*. In **SI.6**, we introduced a new test for this purpose, and we found that the *Nabis* encounter sequence is not random (*P* = 0.009). The Manly preference index (*α*) supports these results. The value of *α* indicates preference when it exceeds 0.5, rejection when it is lower than 0.5 and indifference when it is exactly 0.5. In our trial, *Nabis* exhibited a clear preference for *S*. *exigua* larvae (heterospecific prey; *α*_1_ = 0.65 ± 0.14) and rejection of the conspecific nymphs (*α*_2_ = 0.35 ± 0.14; Wilcoxon test *P* = 0.009).

### Summary

Although *Nabis* exhibits more complex behaviour than our theoretical model, we found that the cannibalistic *Nabis* uses a search image but not with absolute intention because it can also encounter non-desired prey types; however, the its encounter sequence is not determined by the preys’ densities, i.e., *Nabis* encounters its preferred prey with a higher probability than its potential conspecific prey. This finding corroborates results on the subject that have been published elsewhere^29^.

## Discussion

Some insight into the use of a search image may be useful from both the theoretical and applied ecological perspectives. In theoretical ecology, one of the possible mechanisms for maintaining diversity is negative frequency-dependent selection, i.e., rare prey experience higher survival than more common types. Search image formation has been invoked as a possible proximate explanation for this mechanism^43^. For instance, Bond and Kamil^44^ found that apostatic selection by blue jays produces balanced polymorphism in virtual prey.

Although we concentrate on *Nabis*, our theoretical model provides some general insight. First, because the numerical response reaches its maximum at the maximum energy intake, our results are also valid for cases in which reproduction time constraint has no effect on the foraging process. Thus, our result that an **SIU** does not necessarily collect more food than a **RS** is valid in general. Consequently, our hypothesis that an optimal forager must use mixed behaviour, i.e., either acts as an intentional search image user or an opportunistic random searcher (but only one at a time), according to the density of its prey should be tested.

Another possibility is that **SIUs** are not purely intentional (i.e., if they reaches their preferred prey type with a probability less than one). As we found, *Nabis* falls into this category because its encounter sequence is not randomly determined by prey densities. In this case, opportunism is also possible.

For an overview, we note that the mechanism of our theoretical model might also be applied in the contexts of other situations of food choice and is also relevant for the biological control of pests when performed with a significant number of predatory species^31,45,46^. Moreover^47^, such predators may be classified according to their diet or by their role in ecological food webs as “predators” or “true omnivores”. In turn, the former may be specialist and generalist predatory species^48^. Generalist arthropod predators are typically bitrophic; they simultaneously occupy the third and fourth trophic levels by feeding on both herbivores and each other^28^. Moreover, most generalist predators are cannibals^49^. In turn, true omnivorous arthropods feed on both herbivores and plants^46^.

According to the above observation, and in relation to biological control, the results found in the present work can be considered in one predator-two prey systems in two situations: (i) a one omnivorous predator-two prey situation and (ii) a situation with a generalist predator that exhibits cannibalism; this situation would be a one generalist predator-two prey system (i.e., conspecific and heterospecific prey).

The first assumption can be represented by two events. If the true omnivorous *Nesidicoris tenuis* (Reuter) (Hemipera, Miridae) and their prey *Bemisia* tabaci (Gennadius) (Hemiptera, Aleyrodidae) and *Tuta absoluta* (Meyrick) (Lepidoptera: Gelechiidae) are present in greenhouse tomato crops, when both pest species are present in the crop, the biological control of the second species is poor^50^. Another example is represented by the true omnivorous *Macrolophus pygmaeus* (Rambur) (Hemiptera, Miridae) in the same conditions with pest species^51,52^. In the second case, a generalist predator that exhibits cannibalism can be represented by the species studied here in the experimental part, i.e., *N*. *pseudoferus*. In this case, it has been demonstrated that, in the presence of conspecifics, adult females are **SIUs**, which results in less efficient biological control of the pest species^29^ as has been demonstrated in other studies^53^. Similar results have been reported in relation to cannibalism in the case of the general predator mite *Typhlodromus pyri* Schueten (Acari: Phytoseiidae), which are agents that are used against tetranychid pest mites of apples^54^. All of the above references can only be explained if omnivores are **SIUs** instead of **RSs**.

The main property of the search image^7^ is that the prey preference of the forager does affect the encounter probabilities for its prey types. Thus, if an encounter sequence is known, the application of the methodology proposed here enables the determination of whether the predator uses a search image. We hope our model and methodology will be useful in the study of human visual foraging^55–58^ because, in human experiments, a clear distinction should be made between observing a target (a fixated gaze should correspond to encounter) and consumption (finger tapping should correspond to a kill)^59^.

In summary, the **SIU** approach versus the **RS** approach seems to be one of the crucial factors that should be considered if omnivorous and generalist species are used for biological control in agricultural ecosystems. Especially, as mentioned above, because of the current trend of biological control that consists of the use of generalist predatory species and, even more vigorously, in cases involving omnivorous species.

## Methods

In *theoretical part*, we use mathematical tools.

### Experimental trial

The trial methodology was adapted from previous work^16,29^. Mated *N*. *pseudoferus* adult females were used less than one week after the final nymphal ecdysis. They were individually isolated in Petri dishes and subjected to a starving period of 24 h prior to testing. The subjects were given a piece of sponge moistened with distilled water. Six specimens of second-instar *S*. *exigua* larvae were utilized as heterospecific prey, and six specimens of second-instar *N*. *pseudoferus* nymphs were utilized as conspecific prey; the prey were introduced into a choice arena (Petri dish), and then a single *N*. *pseudoferus* adult female was also introduced. Each adult female predator was left to prey on the larvae and nymphs for a period of 4 h. Fifteen replicates were performed for each treatment. Two types of data were recorded: a) the number of prey killed was annotated at the end of the trial (4 h), and b) the prey-capture sequence of adult females was also recorded. Because direct human observation may interfere with the predation behaviour of *Nabis* species^60^, we photographed the trial arena every 10 seconds using an Eos 550D (Canon® Inc, Tokyo 146-8501, Japan) digital camera with an EFS 18–55 lens with macro function (Canon®) that was connected with a cable to a computer. The Communication Software for the Camera EOS Utility, version 2.14 was used^61^. The photographs were collected in a time-lapse manner using the Image-Processing and Analysis in Java (ImageJ) software, version 1.49^62^, which recorded the identity of the killed prey and the sequence of the predation events. The adult predators’ preferences for the different offered prey were quantified with the Manly preference index (*α*)^63^. As established by Cock^64^, the Manly index is the only method that accounts for the reduction in prey density that occurs during the course of the trial as has been corroborated in the review by Sherratt and Harvey^65^. The index equation is as follows:

$${\alpha }_{i}=\frac{\frac{{r}_{i}}{{N}_{i}}}{\frac{{r}_{i}}{{N}_{i}}+\frac{{r}_{j}}{{N}_{j}}},$$

where *r*_*i*_ = number of prey *i* consumed, *r*_*j*_ = number of prey *j* consumed, *N*_*i*_ = number of prey *i* offered, and *N*_*j*_ = number of prey *j* offered. Comparisons of the preference indexes were performed using the Wilcoxon signed-rank test.

## Electronic supplementary material

Supplementary information
