# Supplementary material for: Opportunistic random searcher versus intentional search image user
Source: Sci Rep. 2018 Feb 20;8:3336. doi: 10.1038/s41598-018-21563-y (PMC5820282; doi:10.1038/s41598-018-21563-y)
Supplement: Supplementary file 1 — Supplementary information [file 41598_2018_21563_MOESM1_ESM.doc]

**Supplementary Information**

**to the paper**

**Opportunistic random searcher versus intentional search image user**

József Garay

Zoltán Varga,

Tamás F. Móri,

Inmaculada López,

Manuel Gámez,

Juan R. Gallego,

and

Tomás Cabello

The Supplementary Information consists of two main parts. The first one contains the details of the theoretical investigation, the second one is devoted to the description of the experimental study of *Nabis pseudoferus* (Insecta: Hemiptera: Nabidae). The Main Text will be referred to as **MT**.

**I. Theoretical part**

**Main biological assumptions on the predation process**

In a habitat of area *M* there are *H* perception ranges (PR-s), *x* is the number of A-prey, *y* the number of B-prey, and we suppose that$x+y<H$. Furthermore, the habitat is homogeneous i.e. in all PR-s predator-prey interactions are the same.

**Behaviour of prey**

**Assumption 1**. For the sake of simplicity, we have assumed that the prey have no anti-predator behaviour, thus the prey has no effect on the predation process, so we will have an optimal foraging model, where predator maximizes its numerical response at every moment.

**Assumption 2.** Each perception range contains at most one prey, so prey types are randomly separated in the perception ranges in the home range of the predator. Accordingly to this assumption, there are three different types of PR: a PR is either empty, or includes either one A-prey or one B-prey. Let us denote these types by PR:E, PR:A and PR:B, respectively. Symbol PR:X will also be used for the activity of finding type PR:X (X=E, A, B). Consequently, the distribution of PR types is

| PR type | PR:E | PR:A | PR:B |
| --- | --- | --- | --- |
| Probability | $1-\frac{y}{H}-\frac{x}{H}$ | $\frac{x}{H}$ | $\frac{y}{H}$ |

**Behaviour of predator.** The predator has territory, so there is no interaction between two predators during hunting. We consider an optimal forager predator, which actively searches for its desired prey type.

**Assumption 3.** The searching processes of the predator and the distribution of prey are independent.

**Assumption 4.** The traveling time for a prey depends on the density of the prey, it is longer at lower prey density.

**Assumption 5.** There is a stationary distribution of perception range types PR:X (X=E, A, B), which does not change during time *T*, say one day.

**SI.1. What is the numerical response, if the reproduction (laying eggs) also needs time, not only food?**

The functional response is given for a short time duration *T*, while the density changes by predation can be ignored. (E.g. *Nabis* is active during daylight, i.e. *T*≈12 hours). In this section we consider a female predator laying eggs. However, during this time period *T*, the predator either predates (*T_P_* denotes the total time duration of predation during *T*), or lays eggs (*T_E_* is the total time duration of laying eggs during *T*).

**Assumption 6.** We consider the case when reproduction and predation exclude each other, we have

$T=T_{P}+T_{E}$.

The number of eggs, however, also depends on the collected energy.

**Assumption 7.** The numerical response is determined by the energy balance. Below we calculate how the numerical response depends on the intensity of the energy intake. Suppose that the cost of living of the female predator in unit time is *E_CL_*, and the energy cost of one egg is *E_E_* (also including the cost of searching for a place appropriate for the egg and the energy cost of egg laying). Given a foraging strategy described by a vector **s**, let $E(\mathbf{s})$ be the energy intake in unit time of predation, $E_{T}(\mathbf{s})$ the total energy intake from both prey types during time *T*, $W(\mathbf{s})$the number of eggs laid in unit time, i.e. the numerical response, $W_{T}(\mathbf{s})$ the total number of eggs laid during time *T*, and $t_{E}$ the time duration of laying one egg, $t_{E}>1$. Then, the energy balance for the time period *T* is

$W_{T}(\mathbf{s})E_{E}=E(\mathbf{s})T_{P}-E_{CL}T=E(\mathbf{s})(T-TW(\mathbf{s})t_{E})-E_{CL}T=T[E(\mathbf{s})(1-W(\mathbf{s})t_{E})-E_{CL}]$,

implying

$W(\mathbf{s})E_{E}=\frac{W_{T}(\mathbf{s})}{T}E_{E}=E(\mathbf{s})(1-W(\mathbf{s})t_{E})-E_{CL}$.

Hence, for the numerical response corresponding to strategy $\mathbf{s}$, we obtain

$W(\mathbf{s})=\frac{E(\mathbf{s})-E_{CL}}{E(\mathbf{s})t_{E}+E_{E}}$. (1)

It is easy to see, that the numerical response is a strictly increasing function of the energy intake in unit time of predation, as it should obviously be. In particular, the numerical response and the functional response will take their maxima at the same strategy.

**SI.2. Searching time and travelling time**

**Assumption 8.** Whatever the PR type the searching predator finds, it will be the nearest one from that type.

Now the searching time has two components: the first one is travelling time $\tau_{T}$, that may also depend on the density of A-prey and B-prey, the second one is local searching time $\tau_{LS}$ in the PR. For the sake of simplicity we assume that $\tau_{LS}$ does not depend on what the focal PR contains. So we have

$\tau_{S}=\tau_{T}+\tau_{LS}$. (2)

Next we calculate the average distance between the nearest PR:X and the predator.

Consider a *d*-dimensional bounded Borel set $B\subset\text{R}^{d}$ with non-empty interior, and let $Q\in\mathrm{int}B$ be an inner point of *B*. Let *B* be blown up (i.e., uniformly scaled) from *Q* by factor $\theta$. A predator is placed at *Q*. After that *n* uniformly distributed random points (prey) are thrown into *B*, independently of each other. Finally, let $\Delta=\Delta_{\theta, n}$ denote the (random) distance of the predator from the nearest prey.

**Theorem.** *Suppose* $\theta$ *and n tend to infinity in such a way that the number of points, divided by the volume (Lebesgue measure) of the enlarged set, converges to some positive number $\lambda$. Then*

*holds for every positive real number* *k*.

**Proof.**  In fact, we will prove more.

Let $V\left( . \right)$ denote the *d*-dimensional volume (Lebesgue measure) of the Borel set in parentheses. Let $S_{r}$ denote the closed ball with centre *Q* and radius *r*. It is well known that $V\left(S_{r}\right)=C_{d}r^{d}$, where . We will show the following facts.

(i) $\Delta_{\theta, n}\to\xi$ in distribution, where $P\left(\xi>t\right)=\exp\left(-\lambda C_{d}t^{d}\right), t\geq0$. That is, $\xi$ has Weibull distribution [2] with shape parameter *d* and scale parameter $\left(\lambda C_{d}\right)^{-1/d}$.

(ii) $\limsup_{\theta, n\to\infty} E\left(\exp\left(\varepsilon\Delta_{\theta, n}^{d}\right)\right)<+\infty$ for every $\varepsilon>0$ being sufficiently small.

Fact (i) will imply that $h(\Delta_{\theta, n})\to h(\xi)$ in distribution for every continuous function $h:\text{R}\to\text{R}$. By the de la Vallée-Poussin theorem [4, Theorem T22], from (ii) it follows that $\left\{ h(\Delta_{\theta, n})\right\}$ is uniformly integrable provided

(iii) $\lim_{x\to\infty} \exp(-\varepsilon x^{d})h(x)=0$ for every $\varepsilon>0$.

Hence, for every continuous function *h* satisfying (iii) it follows that

$\lim_{\theta, n\to\infty} Eh(\Delta_{\theta, n})=Eh(\xi)$.

Applying this to $h(x)=x^{k}$ we get our Theorem proved. Indeed,

$E\xi^{k}=\int_{0}^{+\infty} kt^{k-1}P(\xi>t) \text{d}t=\int_{0}^{+\infty} kt^{k-1}\exp\left(-\lambda C_{d}t^{d}\right) \text{d}t$.

Substituting $x=t^{d}$ we obtain

as claimed.

Let us continue with the proofs of (i) and (ii). Without loss of generality we can suppose that *Q* is the origin. Let $S_{r}\subset B\subset S_{R}$, $0<r<R<+\infty$. Then we clearly have

$P(\Delta>t)=\left(1-\frac{V\left(S_{t}\right)}{V(\theta B)}\right)^{n}=\left(1-\frac{C_{d}}{V(B)}\left(\frac{t}{\theta}\right)^{d}\right)^{n}\leq\exp\left(-\frac{C_{d}}{V(B)}\left(\frac{t}{\theta}\right)^{d}n\right)$**,** if $0\leq t\leq\theta r$,

$P(\Delta>t)\leq P(\Delta>\theta r)\leq\exp\left(-\frac{C_{d}r^{d}}{V(B)} n\right)$, if $\theta r<t<\theta R$,

$P(\Delta>t)=0$, if $t\geq\theta R$.

By supposition, $n=\lambda V(B)\theta^{d}\left(1+o(1)\right)$; therefore

$\lim_{\theta, n\to\infty} \left(1-\frac{C_{d}}{V(B)}\left(\frac{t}{\theta}\right)^{d}\right)^{n}=\exp\left(-\lambda C_{d}t^{d}\right)$

for every fixed *t*, thus proving (i).

On the other hand,

$\begin{matrix} E\left(\exp\left(\varepsilon\Delta_{\theta, n}^{d}\right)\right)=\int_{0}^{+\infty} \varepsilon d t^{d-1}\exp(\varepsilon t^{d})P(\Delta>t) \text{d}t \\ \leq\int_{0}^{+\infty} \varepsilon d t^{d-1}\exp(\varepsilon t^{d})\exp\left(-\frac{C_{d}}{V(B)}\left(\frac{t}{\theta}\right)^{d}n\right) \text{d}t+\exp\left(-\frac{C_{d}r^{d}}{V(B)} n\right) \int_{\theta r}^{\theta R} \varepsilon d t^{d-1}\exp(\varepsilon t^{d}) \text{d}t . \end{matrix}$ (3)

By supposition, $n=\lambda V(B)\theta^{d}\left(1+o(1)\right)$; consequently, the first integral on the right-hand side of (3) can be estimated by

$\int_{0}^{+\infty} \varepsilon d t^{d-1}\exp\left(-(\lambda C_{d}-\varepsilon+o(1))t^{d}\right) \text{d}t=\int_{0}^{+\infty} \varepsilon\exp\left(-(\lambda C_{d}-\varepsilon+o(1))x\right) \text{d}x$,

which converges to $\frac{\varepsilon}{\lambda C_{d}-\varepsilon}$ if $\varepsilon<\lambda C_{d}$.

As to the second term on the right-hand side of (3), it can be treated as follows.

=$\left[\exp\left(\varepsilon(\theta R)^{d}\right)-\exp\left(\varepsilon(\theta r)^{d}\right)\right] \exp\left(-\frac{C_{d}r^{d}}{V(B)} n\right)\leq\exp\left(\varepsilon(\theta R)^{d}-\lambda C_{d}(\theta r)^{d}(1+o(1))\right)$.

This tends to zero provided $\varepsilon<\lambda C_{d}r/R$. Thus, (ii) is proved. ■

**Background**. In the limit we obtain a Poisson field (homogeneous spatial Poisson process) with intensity $\lambda$ [3, pp. 281–292]. This is characterized by the following four properties.

- The numbers of points falling into disjoint Borel sets are independent random variables.
- The distribution of the number of points falling into a Borel set *D* only depends on the volume of *D*, but is independent of the shape of *D*.
- Points do not stick, that is, the probability that an infinitesimally small set contains at least two points is negligible with respect to the probability that it contains at least one point.
- The mean number of points in a set of unit volume is equal to $\lambda$.

From all these it can be derived that the distribution of the number of points in a set of volume *V* is Poisson$\left(\lambda V\right)$. Hence, $P\left(\Delta>t\right)=\exp\left(-\lambda V(S_{t})\right)=\exp\left(-\lambda C_{d}t^{d}\right)$; in other words, the distribution of is Weibull$\left(d, (\lambda C_{d})^{-1/d}\right)$. This gives back the limits we have computed above.

In particular, if type PR:X has density *λ*_X_ (X=E,A,B), and is randomly distributed, then the average distance between the nearest PR:X and the predator is the following: In one dimension (with a predator moving along a straight-line) it is $\frac{1}{2\lambda}_{X}$, in two dimensions (predator moving along in a plane) it is $\frac{1}{2\lambda_{X}^{ 1/2}}$, while in 3 dimensions it is .

We emphasize that the dimension of the travelling mode of the predator has an important effect on the functional response. (To our knowledge, we are the first to consider this kind of dependence.)

**Types of predator.** We will compare the numerical responses of two types of predators. The first one cannot use a search image, it visits the nearest perception range and the random distributions of prey imply random encounters. This predator has a conditional strategy: having encountered a prey type, the predator either kills or ignores it. This type of predator will be called random searcher, **RS**. The second type of predator uses a search image and finds its desired prey type with a high, density independent probability. For the sake of simplicity we assume this predator finds its preferred prey with probability 1. This type of predator will be called search image user, **SIU**.

**SI.3. Opportunistic random searcher using no search image**

A **RS** looks for the nearest PR, and no matter what it finds, the searching time is the same $\tau_{S}$, calculated from equality (2) with travelling time $\tau_{T}=\frac{1}{v2\lambda_{PR}^{ 1/2}}$ (for planar movement, where *v* is the mean velocity of the predator). Here $\lambda_{PR}=\frac{H}{M}$ is the PR density, *M* is the focal area and for simplicity we take *v*=1*.*

In the table below, similarly to the notation PR:X, symbols K:X and K:NX mean that the predator kills and does not kill an X-prey with probability $s_{A}$ and $s_{B}$, respectively, $\tau_{X}$ is the corresponding predation time and $c_{X}$ denotes the energy intake from an X-prey (X=A,B).

**Table 1**. Activity distribution, activity times and energy intake for a **RS**

| Activity | Probability | Time | Energy intake |
| --- | --- | --- | --- |
| 1. PR:E | $p_{1}=1-\frac{y}{H}-\frac{x}{H}$ | $\tau_{1}=\tau_{S}$ | 0 |
| 2. PR:A; K:A | $p_{2}=\frac{x}{H}s_{A}$ | $\tau_{2}=\tau_{S}+\tau_{A}$ | $c_{A}$ |
| 3. PR:A; K:NA | $p_{3}=\frac{x}{H}(1-s_{A})$ | $\tau_{3}=\tau_{S}$ | 0 |
| 4. PR:B; K:B | $p_{4}=\frac{y}{H}s_{B}$ | $\tau_{4}=\tau_{S}+\tau_{B}$ | $c_{B}$ |
| 5. PR:B; K:NB | $p_{5}=\frac{y}{H}(1-s_{B})$ | $\tau_{5}=\tau_{S}$ | 0 |

Then the average time of one round is

$T(s_{A},s_{B})=\tau_{S}+\frac{x}{H}s_{A}\tau_{A}+\frac{y}{H}s_{B}\tau_{B}$,

By our derivation method [1], for the functional response for the A-prey we get

$F_{A}(s_{A},s_{B}):=\frac{\frac{x}{H}s{}_{A}}{T(s_{A},s_{B})}$,

and similarly, the functional response for the B-prey is

$F_{B}(s_{A},s_{B}):=\frac{\frac{y}{H}s{}_{B}}{T(s_{A},s_{B})}$.

Now, the average energy intake rate per unit time, $E_{R}(s_{A},s_{B})$, is defined as

$E_{R}(s_{A},s_{B}):=\frac{c_{A}\frac{x}{H}s{}_{A}+c_{B}\frac{y}{H}s{}_{B}}{T(s_{A},s_{B})}$.

**RS** as an optimal forager maximizes its energy intake in unit time, so for fixed *x* and *y*, optimal forager adopts strategies $s_{A}^{*}\in\left[0,1\right]$ and $s_{B}^{*}\in\left[0,1\right]$ that maximize its average energy intake,

$E_{R}(s_{A}^{*},s_{B}^{*})\mathop{=\max}\limits_{s_{A},s_{B}} E_{R}(s_{A},s_{B})$. (3)

At the optimal strategy $(s_{A}^{*},s_{B}^{*})$, the numerical response is

$W_{R}(s_{A}^{*},s_{B}^{*})=\frac{E_{R}(s_{A}^{*},s_{B}^{*})-E_{CL}}{E_{R}(s_{A}^{*},s_{B}^{*})t_{E}+E_{E}}$.

As we have mentioned above, see note after equality (1), the numerical response will take its maximum at the same strategy choice as the average energy intake $E_{R}(s_{A}^{*},s_{B}^{*})$. Therefore we will maximize the energy intake rate

$E_{R}(s_{A},s_{B})=\frac{c_{A}\frac{x}{H}s{}_{A}+c_{B}\frac{y}{H}s{}_{B}}{\tau_{S}+\frac{x}{H}s_{A}\tau_{A}+\frac{y}{H}s_{B}\tau_{B}}$,

with respect to the strategy $(s_{A},s_{B})$. First, for fixed $s_{B}$ we get

$\frac{\partial}{\partial s{}_{A}}E_{R}(s_{A},s_{B})=\frac{x}{H}\frac{c_{A}\tau_{S}+\frac{y}{H}s_{B}\left(c_{A}\tau_{B}-c_{B}\tau_{A}\right)}{\left(\tau_{S}+\frac{x}{H}s_{A}\tau_{A}+\frac{y}{H}s_{B}\tau_{B}\right)^{2}}$,

thus

$\mathrm{sgn}\frac{\partial}{\partial s{}_{A}}E_{R}(s_{A},s_{B})=\mathrm{sgn}[c_{A}\tau_{S}+\frac{y}{H}s_{B}\left(c_{A}\tau_{B}-c_{B}\tau_{A}\right)]$.

Similarly to the standard optimal foraging theory, the above sign does not depend on $s_{A}$. Assume that A-prey is more valuable than prey B, formally $\frac{c_{A}}{\tau_{A}}>\frac{c_{B}}{\tau_{B}}$, then $s_{A}^{*}=1$ for all possible fixed $s_{B}$.

Similarly, for fixed $s_{A}^{*}=1$ we get

$\frac{\partial}{\partial s{}_{B}}E_{R}(s_{A},s_{B})=\frac{y}{H}\frac{c_{B}\tau_{S}+\frac{x}{H}\left(c_{B}\tau_{A}-c_{A}\tau_{B}\right)}{\left(\tau_{S}+\frac{x}{H}s_{A}\tau_{A}+\frac{y}{H}s_{B}\tau_{B}\right)^{2}}$,

thus

$$\mathrm{sgn}\frac{\partial}{\partial s{}_{B}}E_{R}(s_{A},s_{B})=\mathrm{sgn}[c_{B}\tau_{S}+\frac{x}{H}\left(c_{B}\tau_{A}-c_{A}\tau_{B}\right)]$$

Consequently

$$s_{B}^{*}=\left\{\begin{matrix} 0\text{ , if }x>\frac{c_{B}\tau_{S}}{c_{A}\tau_{B}-c_{B}\tau_{A}}H \\ 1,\text{ if }x<\frac{c_{B}\tau_{S}}{c_{A}\tau_{B}-c_{B}\tau_{A}}H. \end{matrix}\right.$$

We will use the following notation $x_{R}:=\frac{c_{B}\tau_{S}}{c_{A}\tau_{B}-c_{B}\tau_{A}}H$, which defines the vertical straight line (switching line for **RS**), corresponding to $\sigma_{R}$ in Figure 1 of **MT**.

**One-dimensiononal case.** For further calculations let us assume that **RS** moves in one dimension, thus we have $\tau_{S}=\tau_{T}+\tau_{LS}=\frac{1}{2\lambda}_{X}\text{ }+\tau_{LS}=\frac{1}{2\frac{H}{M}}+\tau_{LS}=\frac{M}{2H}+\tau_{LS}$, so

$$s_{B}^{*}=\left\{\begin{matrix} 0,\text{ if }x>\frac{c_{B}\left(\frac{M}{2H}+\tau_{LS}\right)}{c_{A}\tau_{B}-c_{B}\tau_{A}}H \\ 1,\text{ if }x<\frac{c_{B}\left(\frac{M}{2H}+\tau_{LS}\right)}{c_{A}\tau_{B}-c_{B}\tau_{A}}H, \end{matrix}\right.$$

and $x_{R}:=\frac{c_{B}(\frac{M}{2H}+\tau_{LS})}{c_{A}\tau_{B}-c_{B}\tau_{A}}H$.

**SI.4. Intentional search image user SIU**

Now we will consider a **SIU** finding its nearest desired prey with probability 1. So, we have two density dependent travelling times corresponding to the desired prey: $\tau_{TA}$ and $\tau_{TB}$. In fact, let us suppose first that the **SIU** searching for an A-prey, found an A-prey with probability 1, then it must be the nearest one among the A-preys, for which we have $\lambda_{A}=\frac{x}{M}$. Similarly, for the nearest PR with a B-prey we have $\lambda_{B}=\frac{y}{M}$. Applying the Theorem of section SI.2, we can calculate the traveling time of the **SIU**.

In other words, a **SIU** can find no empty PR and it has two searching modes: when looking for an A-prey then it cannot encounter a B-prey and vice versa. Thus **SIU** has only a one-dimensional optimal foraging strategy, looking for an A-prey with probability *s*, and for a B-prey with probability 1-*s*.

Next we will calculate the functional response of this type of predator having the following activity distribution:

**Table 2.** Activity distribution, activity times and energy intake for a **SIU** searching for A-prey with probability *s*

| Activity | Probability | Time | Energy intake |
| --- | --- | --- | --- |
| Looking for prey A | $s$ | $t_{A}=\tau_{TA}+\tau_{LS}+\tau_{A}$ | $c_{A}$ |
| Looking for prey B | $1-s$ | $t_{B}=\tau_{TB}+\tau_{LS}+\tau_{B}$ | $c_{B}$ |

For strategy $s\in\left[0,1\right]$, the average activity time is

$T_{I}(s):=st_{A}+(1-s)t_{B}$.

So, for fixed strategy $s$, applying our derivation method [1], the functional responses of a **SIU** searching for A-prey and B-prey, respectively, are

$\Phi_{A}(s):=\frac{s}{st_{A}+(1-s)t_{B}}$, and $\Phi_{B}(s):=\frac{1-s}{st_{A}+(1-s)t_{B}}$.

Thus, for the average energy intake in unit time, we obtain

$E_{I}(s):=c_{A}\Phi_{A}(s)+c_{B}\Phi_{B}(s)$.

**SIU**, as an optimal forager, maximizes its energy intake in unit time, so for fixed *x* and *y*, it will choose an optimal strategy $s^{*}\in\left[0,1\right]$ that maximizes its energy intake, formally

$E_{I}(s^{*})=\max_{s} E_{I}(s)$. (4)

At the optimal strategy $s^{*}$, the numerical response is

$W_{I}(s^{*})=\frac{E_{I}(s^{*})-E_{CL}}{E_{I}(s^{*})t_{L}+E_{E}}$.

As we have already seen, functions $W_{I}$ and $E_{I}$ take their optima at the same strategy $s^{*}$, so we will maximize the average energy intake function

$E_{I}(s)=\frac{c_{A}s+c_{B}(1-s)}{st_{A}+(1-s)t_{B}}$,

where $t_{A}=\tau_{TA}+\tau_{LS}+\tau_{A}$ and $t_{B}=\tau_{TB}+\tau_{LS}+\tau_{B}$. From

$\frac{\partial}{\partial s}E_{I}(s)=\frac{c_{A}t_{B}-c_{B}t_{A}}{\left(st_{A}+(1-s)t_{B}\right)^{2}}$,

we get

$$s^{*}=\left\{\begin{matrix} 0,\text{ if }\frac{c_{A}}{t_{A}}<\frac{c_{B}}{t_{B}} \\ 1,\text{ if }\frac{c_{A}}{t_{A}}>\frac{c_{B}}{t_{B}}. \end{matrix}\right.$$

Here $\frac{c_{A}}{t_{A}}>\frac{c_{B}}{t_{B}}$ means $\frac{c_{A}}{\tau_{TA}+\tau_{LS}+\tau_{A}}>\frac{c_{B}}{\tau_{TB}+\tau_{LS}+\tau_{B}}$. Observe that $\tau_{TA}$ and $\tau_{TB}$ depend on the density of A-prey and B-prey, respectively, so the density dependent values of the A-prey and B-prey determine the strategy of the **SIU**. In other words, if $\frac{c_{A}}{t_{A}}>\frac{c_{B}}{t_{B}}$, the intentional predator only eats A-prey and if a $\frac{c_{A}}{t_{A}}<\frac{c_{B}}{t_{B}}$, it only eats B-prey. Furthermore, we say that A-prey is *locally more valuable* than B-prey, if $\frac{c_{A}}{\tau_{LS}+\tau_{A}}>\frac{c_{B}}{\tau_{LS}+\tau_{B}}$, and *more valuable in the usual sense,* if $\frac{c_{A}}{\tau_{A}}>\frac{c_{B}}{\tau_{B}}$.

We emphasize that the **SIU** follows again the zero-one rule, but it can find its desired prey with probability 1, so it has no option to be opportunistic, since if it is looking for B-prey it cannot encounter with a more valuable A-prey. As we have seen, travelling times $\tau_{TA}$ and $\tau_{TB}$ also depend on the dimension of home rage of the predator. Since in the considered one-dimensional case, by the Theorem of **SI.2,** we have $\tau_{TA}=\frac{M}{2x}$ and $\tau_{TB}=\frac{M}{2y}$, thus the **SIU** is looking for B-prey (i.e. $s^{*}=0$), if $\frac{c_{A}}{t_{A}}<\frac{c_{B}}{t_{B}}$, i.e. if

$\frac{c_{A}}{\frac{M}{2x}+\tau_{LS}+\tau_{A}}<\frac{c_{B}}{\frac{M}{2y}+\tau_{LS}+\tau_{B}}$.

After some calculation we get

$$y>\frac{xMc_{A}}{Mc_{B}+\left(\left(\tau_{LS}+\tau_{A}\right)c_{B}-\left(\tau_{LS}+\tau_{B}\right)c_{A}\right)2x}.$$

Therefore, the equation of the switching curve $\sigma_{I}$ of **SIU** in Figure 1 is

$$y=\frac{xMc_{A}}{Mc_{B}+\left(\left(\tau_{LS}+\tau_{A}\right)c_{B}-\left(\tau_{LS}+\tau_{B}\right)c_{A}\right)2x}.$$

Now the behaviour of the right-hand side depends on the sign of $\left(\tau_{LS}+\tau_{A}\right)c_{B}-c_{A}\left(\tau_{LS}+\tau_{B}\right)$. If A-prey is locally more valuable than B-prey, then $\left(\tau_{LS}+\tau_{A}\right)c_{B}-\left(\tau_{LS}+\tau_{B}\right)c_{A}<0$. Hence the switching curve $\sigma_{I}$ is hyperbolic with vertical asymptote

$x_{I}=\frac{1}{2}\frac{Mc_{B}}{\left(\tau_{LS}+\tau_{B}\right)c_{A}-\left(\tau_{LS}+\tau_{A}\right)c_{B}}$,

which is in the positive half plane $x>0$, since $\left(\tau_{LS}+\tau_{A}\right)c_{B}-\left(\tau_{LS}+\tau_{B}\right)c_{A}<0$.

Furthermore, we note that $x_{I}=\frac{1}{2}\frac{Mc_{B}}{\left(\tau_{LS}+\tau_{B}\right)c_{A}-\left(\tau_{LS}+\tau_{A}\right)c_{B}}<\frac{\tau_{S}c_{B}}{c_{A}\tau_{B}-c_{B}\tau_{A}}H=x_{R}$.

**SI.5. Does the intentional SIU overperform opportunistic RS?**

Now we will compare the considered predator types in terms of the energy intakes per unit time:

$$E_{R}(s_{A},s_{B})=\frac{c_{A}\frac{x}{H}s{}_{A}+c_{B}\frac{y}{H}s{}_{B}}{\frac{M}{2H}+\tau_{LS}+\frac{x}{H}s_{A}\tau_{A}+\frac{y}{H}s_{B}\tau_{B}}$$

and

$E_{I}(s)=\frac{c_{A}s+c_{B}(1-s)}{s(\frac{M}{2x}+\tau_{LS}+\tau_{A})+(1-s)(\frac{M}{2y}+\tau_{LS}+\tau_{B})}$.

It is intuitively clear that in terms of energy intake, **RS** might be better than **SIU,** if **RS** accepts both prey types ($s{}_{A}=s{}_{B}=1$), while **SIU** looks for B-prey only (*s=*0). In terms of the energy intake this means

$E_{R}(1,1)=\frac{c_{A}\frac{x}{H}+c_{B}\frac{y}{H}}{\tau_{T}+\tau_{LS}+\frac{x}{H}\tau_{A}+\frac{y}{H}\tau_{B}}=\frac{c_{A}\frac{x}{H}+c_{B}\frac{y}{H}}{\frac{M}{2H}+\tau_{LS}+\frac{x}{H}\tau_{A}+\frac{y}{H}\tau_{B}}>\frac{c_{B}}{\tau_{TB}+\tau_{LS}+\tau_{B}}=\frac{c_{B}}{\frac{M}{2y}+\tau_{LS}+\tau_{B}}=E_{I}(0)$.

The (*x*,*y*) regions defined by inequalities $E_{R}(1,1)>E_{I}(0)$ and $E_{R}(1,1)<E_{I}(0)$, are separated by the curve defined by equation $E_{R}(1,1)=E_{I}(0)$, i.e. by

$\frac{c_{A}\frac{x}{H}+c_{B}\frac{y}{H}}{\frac{M}{2H}+\tau_{LS}+\frac{x}{H}\tau_{A}+\frac{y}{H}\tau_{B}}=\frac{c_{B}}{\frac{M}{2y}+\tau_{LS}+\tau_{B}}$.

Hence the equation of the curve corresponding to $\gamma_{1}$ of Figure 2 of **MT** is

$x=\frac{2\tau_{LS}c_{B}y(H-y)}{Mc_{A}+(c_{A}\tau_{LS}+c_{A}\tau_{B}-c_{B}\tau_{A})2y}$.

Similarly to the above reasoning, **RS** might be better than **SIU,** if **RS** accepts both prey types ($s{}_{A}=s{}_{B}=1$), while **SIU** looks for A-prey only (*s=*1). Then, in terms of the energy intake we have

$$E_{R}(1,1)=\frac{c_{A}\frac{x}{H}+c_{B}\frac{y}{H}}{\tau_{T}+\tau_{LS}+\frac{x}{H}\tau_{A}+\frac{y}{H}\tau_{B}}=\frac{c_{A}\frac{x}{H}+c_{B}\frac{y}{H}}{\frac{M}{2H}+\tau_{LS}+\frac{x}{H}\tau_{A}+\frac{y}{H}\tau_{B}}>\frac{c_{A}}{\tau_{TA}+\tau_{LS}+\tau_{A}}=\frac{c_{A}}{\frac{M}{2x}+\tau_{LS}+\tau_{A}}E_{I}(1)$$

Now the equation of the curve corresponding to $\gamma_{2}$ of Figure 2 of **MT** is

$y=\frac{2\tau_{LS}c_{A}x(H-x)}{Mc_{B}+(c_{B}\tau_{LS}+c_{B}\tau_{A}-c_{A}\tau_{B})2x}$.

We note that the simulations for Figures 1-3 of **MT** have been programmed in Matlab environment, with parameter values:$H:=4000$, $M:=10000$, $c_{A}:=1$, $c_{B}:=2$, $\tau_{LS}:=1$, $\tau_{A}:=1$, $\tau_{B}:=8$.

Finally, we show that the appearance of the density range where *E_R_*>*E_I_*  is a phenomenon robust against the change of key parameters$c_{B}$ and $\tau_{B}$. First we show that, if the gap between the values of A-prey and B-prey is large enough, namely $\frac{c_{B}}{\tau_{B}}<\frac{4}{13}$, then curves $\gamma_{1}$ and $\gamma_{2}$ display a pattern similar to Figure 2 of **MT**. Indeed, with parameters $H:=4000$, $M:=10000$, $c_{A}:=1$, $\tau_{LS}:=1$, $\tau_{A}:=1$, the equation of curve $\gamma_{2}$ is $y=\frac{2x(4000-x)}{10000c_{B}+(c_{B}+c_{B}-\tau_{B})2x}$.

An easy calculation shows that, for $\frac{c_{B}}{\tau_{B}}<\frac{4}{13}$, $x_{0}=\frac{5000c_{B}}{\left(\tau_{B}-2c_{B}\right)}$ defines a vertical asymptote of $\gamma_{2}$, with $x_{0}<4000=H$, where *H* is the number of perception ranges in our example. Therefore, under the condition $\frac{c_{B}}{\tau_{B}}<\frac{4}{13}$, curves $\gamma_{1}$ and $\gamma_{2}$ display a pattern similar to Figure 2 of **MT**. Furthermore, in this case, measuring the size of the density range where *E_R_*>*E_I_* , with the area of the range between $\gamma_{1}$ and $\gamma_{2}$, we find that this area remains strictly positive, as shown in Figure 4 of **MT**. The calculations necessary to plot this area in function of parameters $c_{B}$ and $\tau_{B}$ were realized in MatLab environment.

**II. Experimental part: The existence of searching image**

The specimens used in the test were the following: *Nabis* (*Nabis*) *pseudoferus* Remane (Insecta, Hemiptera, Nabidae), *Spodoptera* *exigua* (Hübner) (Insecta, Lepidoptera, Noctuidae), and *Ephestia* *kuehniella* Zeller (Insecta, Lepidoptera, Phycitinae). The colony of *N. pseudoferus* was established from wild populations at different locations of the Southeast of Spain, and reared under laboratory conditions. We used plastic containers (12 l) equipped with a cardboard panel to act as a shelter material, bean pods as a substrate for oviposition and a water source, and *E. kuehniella* eggs as rearing prey. These eggs were supplied frozen (Ephescontrol®, Agrobio S.L., La Mojonera, Almeria, Spain) and kept at -40 °C until use. The *N. pseudoferus* specimens used in the trial were reared in the laboratory for only two generations before performing the trial. The *S. exigua* larvae used as prey were provided as eggs by the company Entomotech S.L. (Almeria, Spain). After hatching, the larvae were reared on an artificial diet following the methodology described by Cabello et al. [5] up to the second larval stage. The conditions for rearing and conducting the two tests were 25±1 °C and 60-80% HR.

The laboratory arena method was used in the trial. Despite the fact that this method may underestimate the daily consumption of prey in relation to the use of field cages, it is still considered appropriate to use this method to estimate such values [6]). The arenas were Petri dishes (Ø = 9 cm, h = 1.5 cm) sealed with Parafilm© to prevent the escape of S. exigua larvae.

The choice trial was conducted with *N. pseudoferus* adult females, second-instar larvae of *S. exigua* (as heterospecific prey), and second-instar nymphs of *N. pseudoferus* (as conspecific prey) together. Fifteen replicates were carried out for each treatment. The trial methodology was adapted from Cabello et al. [7]. *N. pseudoferus* mated adult females were used less than one week after final nymphal ecdysis. They were individually isolated in Petri dishes and subjected to a starving period of 24 h prior to testing. They were only given a piece of sponge moistened with distilled water. Six specimens of each species were used in each replication. Each adult female predator was left to prey on them for a period of 4 h. The recorded data were the species and order of prey killed by depredatory females. Since direct human observation may interfere with the predation behaviour of *Nabis* species [8]), we photographed the trial arena every 10 seconds using an Eos 550D (Canon®) digital camera, EFS 18-55 lens with macro function (Canon®), connected with a cable to a computer. The software used was Communication Software for the Camera EOS Utility, version 2.14 [9]. Due to the type of lens and pixel resolution, images of only six arenas could be captured simultaneously, and it thus took four days to complete all replicates. Photographs were set in time-lapse using the Image Processing and Analysis in Java (ImageJ) software, version 1.49 [10], which recorded the behaviour, the duration of a predation event, the identity of the killed prey, and the sequence of predation events.

**SI.6. A statistical test for checking preference in a sequence**

We develop a test in the context of the present study, supposing that a certain predator can choose from two types of prey, A and B. We want to know if it has any preference. To test this, individuals

were observed in the presence of different preys, namely, *a* of A-prey and *b* of B-prey. The predator

consumed the prey, one after another, in a certain order. The duration of the test was fixed, so it could happen that not every prey was killed, or better to say, not every kill was observed. During the time of test the animal had $m\leq a+b$ preys, in the order of consummation as follows: $X_{1},X_{2},\ldots,X_{m}$, where $X_{i}=1$ if A-prey, and $X_{i}=-1$ if B-prey was chosen. Even if the test was over before all preys were taken, we can define (but not observe) $X_{i}$ for $i>m$, as well.

Let $S_{k}=X_{1}+\ldots+X_{k}$, then $S_{a+b}=a-b$. If no preference is supposed, every ordering of the preys is equiprobable, and $S_{k}$ varies approximately like $\frac{a-b}{a+b}\cdot k$. On the other hand, if the animal has preference, $S_{k}$ grows or decreases more rapidly in the beginning, until the preferred prey becomes too rare. Hence, the stronger the preference is, the greater values the statistic

$$W=\mathop{\max}\limits_{1\leq k\leq m} \left|S_{k}-\frac{a-b}{a+b}\cdot k\right|$$

tends to take on. Thus, *W* appears a correct indicator of preference. Forgetting about *m* causes bias in the conservative direction, decreasing the size (= level of significance) of the test with respect to its nominal value), hence the rejection of the null hypothesis of no preference still remains informative.

The *p*-value of *W* can be obtained by considering all possible orderings of the preys (there is $\left(\begin{matrix} a+b \\ a \end{matrix}\right)$ of them, which is less than one thousand for $a+b=12$), and counting in what proportion of the orderings will *W* be greater than what was observed.

Finally, we end with a couple of *p*-values, to which we can apply Fisher’s combined probability test: in the absence of preference, the statistic $-2\sum_{i=1}^{N} \ln(p_{i})$ would follow chi-square distribution with 2*N* degrees of freedom (*N* is the number of tests).

Alternatively, we can use Stouffer's *Z*-score method, where it is possible to weight the tests according to the number *m* of kills: greater *m* is rewarded with a greater weight $w_{i}$. The test statistic is

$Z=\frac{1}{\sqrt{D}} \sum_{i=1}^{N} w_{i}\Phi^{-1}(1-p_{i})$, where $D=\sum_{i=1}^{N} w_{i}^{2}$,

and $\Phi$ is the standard normal cumulative distribution function. Assuming no preference *Z* is approximately standard normal, hence a one sided *z*-test can be performed. The Fisher test and the Stouffer method are available with statistical software packages or in the *metap* R-package.

When performing the test attention must be paid to ties. They can cause that though the *p*-values of individual tests do not support our conjecture of prey preference, Fisher’s combined test shows a significant deviation from the null hypothesis of no preference, as it was the case with our data. For example, when *a* = *b* = 6, then the number of all possible orderings is $\left(\begin{matrix} 12 \\ 6 \end{matrix}\right)=924$, but *W* can take on not more than 7 different values. This causes lumps in the distribution of the *p*-value, diverting it from the uniform distribution, even under the null hypothesis of no preference. (Note that the Fisher test checks the uniformity of the given collection of *p*-values.) This may be overcome by adding a small random number to *W* as well as to each of the 924 reference values. This should be repeated for each experiment; besides, the random additive terms of reference values must be renewed in every case, too, otherwise the collection of *p*-values will not be independent any more. (In the general (*a*,*b*) case the additive random terms have to fall in the interval $\left(0,\frac{\gcd(a+b,a-b}{a+b}\right)$, in order to avoid transpositions.) This is quite tiresome. It might be of less work to generate a random rank for the observed *W* as follows. If the observed value of *W* has multiplicity *u* among the reference values, and there are *v* reference values that are greater than our *W*, then the corresponding *p*-value is to be drawn from the set $\left(\frac{v+1}{M},\frac{v+2}{M},...,\frac{v+u}{M}\right)$, where $M=\left(\begin{matrix} a+b \\ a \end{matrix}\right)$, uniformly at random, and independently in each experiment.

**SI.7. Prey preference analysis**

The results of the test carried out on prey preference, as described in section SI.6, are shown in Table 3.

**Table 3.** Order of killing of prey, when 12 prey (6 heterospecific prey *Spodoptera exigua*, and 6 conspecific prey *Nabis pseudoferus*) were exposed to a *N. pseudoferus* predatory female, under laboratory conditions (25±1 °C, 60-80% HR), during 4 hours.

| Replicate | Killed prey (C = conspecific prey, H = heterospecific prey) | | | | | | | | | | | |
| --- | --- | --- | --- | --- | --- | --- | --- | --- | --- | --- | --- | --- |
| No. | 1 | 2 | 3 | 4 | 5 | 6 | 7 | 8 | 9 | 10 | 11 | 12 |
| 1 | H | H | H | C | H | H | H | C | - | - | - | - |
| 2 | C | H | H | H | H | H | - | - | - | - | - | - |
| 3 | H | H | C | C | H | H | - | - | - | - | - | - |
| 4 | C | H | H | C | C | H | - | - | - | - | - | - |
| 5 | C | C | C | H | H | H | H | C | H | H | - | - |
| 6 | C | H | H | H | H | H | C | C | C |  |  |  |
| 7 | C | H | H | H | H | H | H | - | - | - | - | - |
| 8 | C | H | C | H | H | H | H | - | - | - | - | - |
| 9 | C | H | C | H | C | H | - |  |  |  |  |  |
| 10 | C | C | H | H | H | H | H | - | - | - | - | - |
| 11 | C | H | H | C | C | C | H |  |  |  |  |  |
| 12 | C | H | H | H | H | H | C | - | - | - | - | - |

The adult predators’ preferences towards different offered prey were studied using the Manly preference index (α) [11]. As established by Cock [12], the Manly index is the only method that takes into account the reduction in prey density that occurs during the course of the trial. This has been corroborated in the review by Sherratt and Harvey [13]. The index equation is as follows:

,

where *r_i_* = number of prey *i* consumed, *r_j_* = number of prey *j* consumed, *N_i_* = number of prey *i* offered, and *N_j_* = number of prey *j* offered.

The values of this index indicate indifference if equal to 0.5, rejection if below 0.5, and attraction when over 0.5. Comparisons of preference indexes between two types of prey were carried out using the Wilcoxon signed-rank test on paired data.

**References**

[1] Garay, J. & Móri, F. T. When is the opportunism remunerative? *Community Ecol*. **11**, 160-170 (2010).

[2] Grimmett, G. R.; and Stirzaker, D. *Probability and Random Processes*, *3rd ed.*, Oxford University Press (2001).

[3] Johnson, N.; Kotz, S.; and Balakrishnan, N. *Continuous Univariate Distributions, Vol. 2, 2nd ed.* New York: Wiley (1995).

[4] Meyer, P.A. *Probability and Potentials*, New York: Blaisdell Publishing Co (1966).

[5] Cabello T., Rodriguez, H. & Vargas P. Development, longevity and fecundity of *Sopodoptera littoralis* (Lep.: Noctuidae) reared on eight artificial diets. *J. Appl. Entomol*. **97**, 494-499 (1984).

[6] Latham D.R. & Mills N.J. Quantifying insect predation: a comparison of three methods for estimating daily per capita consumption of two aphidophagous predators. *Environ. Entomol*. **38**, 1117-1125 (2009).

[7] Cabello T., Bonfil F., Gallego J.R., Fernandez-Maldonado F.J., Gámez M. & Garay J. 2015. Can interactions between an omnivorous hemipteran and an egg parasitoid limit the level of biological control for the tomato pinworm? *Environ. Entomol*. **44**, 12-26 (2015).

[8] Wade M.R., Zalucki M.P. & Franzmann B.A. Influence of observer presence on Pacific damsel bug behavior: who is watching whom? *J. Insect Behav*. **18**, 651-667 (2005).

[9] Canon. *Communication Software for the Camera EOS Utility*, Version 2.14. Canon Inc. (2014).

[10] Schneider C.A., Rasband W.S. & Eliceiri K.W. NIH Image to ImageJ: 25 years of image analysis. *Nat. Methods* **9**, 671-675 (2012).

[11] Manly B.F.J., Miller P. & Cook L. Analysis of a selective predation experiment. *Am. Nat*. **106**, 719-736 (1972).

[12] Cock M. The assessment of preference. *J. Anim. Ecol*. **47**, 805-816 (1978).

[13] Sherratt T. & Harvey I. Frequency-dependent food selection by arthropods: a review. *Biol. J. Linn. Soc*. **48**, 167-186 (1993).
